# Molecular mapping of the *Pi2/9* allelic gene *Pi2-2* conferring broad-spectrum resistance to *Magnaporthe oryzae* in the rice cultivar Jefferson

**DOI:** 10.1186/1939-8433-5-29

**Published:** 2012-10-03

**Authors:** Nan Jiang, Zhiqiang Li, Jun Wu, Yue Wang, Liqun Wu, Suhua Wang, Dan Wang, Ting Wen, Yi Liang, Pingyong Sun, Jinling Liu, Liangying Dai, Zhilong Wang, Chao Wang, Meizhong Luo, Xionglun Liu, Guo-Liang Wang

**Affiliations:** 1 Hunan Provincial Key Laboratory of Crop Germplasm Innovation and Utilization, College of Agronomy, College of Bio-Safety Science and Technology, Hunan Agricultural University, Changsha, 410128 China; 2 State Key Laboratory for Biology of Plant Diseases and Insect Pests, Institute of Plant Protection, Chinese Academy of Agricultural Sciences, Beijing, 100193 China; 3 State Key Laboratory of Crop Genetic Improvement, Huazhong Agricultural University, Wuhan, 430070 China; 4 Department of Plant Pathology, Ohio State University, Columbus, Ohio 43210 USA

**Keywords:** Rice blast, Resistance gene, Mapping, BAC clones, *Pi2/9* locus

## Abstract

**Background:**

Utilization of broad-spectrum resistance (*R*) genes is an effective and economical strategy to control the fungal pathogen *Magnaporthe oryzae*, the causal agent of the rice blast disease. Among the cloned blast resistance genes, *Pi9*, *Pi2* and *Piz-t* confer broad-spectrum resistance to diverse *M. oryzae* isolates and were isolated from the *Pi2/9* locus on chromosome 6. Identification and isolation of additional *R* genes with different resistance spectra from this locus will provide novel genetic resources for better control of this important rice disease.

**Results:**

In this study, we identified a dominant *R* gene, *Pi2-2,* at the *Pi2/9* locus from Jefferson, an elite U.S. rice cultivar, through genetic and physical mapping. Inoculation tests showed that Jefferson has different resistant specificities to *M. oryzae* isolates compared rice lines with the *Pi9*, *Pi2* and *Piz-t* genes*.* Fine mapping delimited *Pi2-2* to a 270-kb interval between the markers AP5659-3 and RM19817, and this interval contains three nucleotide-binding site-leucine-rich repeat (NBS-LRR) genes in the Nipponbare genome. Five bacterial artificial chromosome (BAC) clones spanning the region were identified, and a BAC contig covering the *Pi2-2* locus was constructed.

**Conclusions:**

We identified a new allelic gene at the *Pi2/9* locus and fine-mapped the gene within a 270-kb region. Our results provide essential information for the isolation of the *Pi2-2* gene and tightly linked DNA markers for rice blast resistance breeding.

**Electronic supplementary material:**

The online version of this article (doi:10.1186/1939-8433-5-29) contains supplementary material, which is available to authorized users.

## Background

Rice is the staple food for more than half people of the world, and the demand is increasing because of the expanding rice-eating population, particularly in many developing countries in Africa and Asia. However, rice production is severely affected by various biotic and abiotic stresses ([Khush and Jena 2009]). Rice blast, caused by the fungal pathogen *Magnaporthe oryzae*, is one of the major limitations, and usually causes 10-30% yield loss in rice production when a rice blast epidemic occurs ([Talbot 2003];[Skamnioti and Gurr 2009]). Use of host resistance is an effective and economical way to control the blast disease ([Khush and Jena 2009]). To date, over 80 blast resistance genes have been identified, and are distributed on 11 rice chromosomes except chromosome 3 ([Liu et al. 2010];[Yang et al. 2009]). So far, 21 have been cloned (*Pib*, *Pita*, *Pi9*, *Pi2*, *Piz-t*, *Pid2*, *Pi36*, *Pi37*, *Pik-m*, *Pit*, *Pi5*, *Pid3*, *pi21*, *Pb1*, *Pish*, *Pik*, *Pik-p*, *Pi54*, *Pia*, *NLS1* and *Pi25*). Interestingly, most of them are NBS-LRR genes except *Pi-d2* and *pi21* ([Wang et al. 1999];[Bryan et al. 2000];[Qu et al. 2006];[Zhou et al. 2006];[Chen et al. 2006];[Liu et al. 2007];[Lin et al. 2007];[Ashikawa et al. 2008];[Hayashi and Yoshida. 2009];[Lee et al. 2009];[Shang et al. 2009];[Fukuoka et al. 2009];[Hayashi et al. 2010];[Takahashi et al. 2010];[Zhai et al. 2011];[Yuan et al. 2011];[Sharma et al. 2005];[Okuyama et al. 2011];[Tang et al. 2011];[Chen et al. 2011]). *Pi-d2* encodes a receptor-like kinase protein with a predicted extracellular domain of a bulb-type mannose-specific binding lectin (B-lectin) and an intracellular serine-threonine kinase domain ([Chen et al. 2006]). *Pi21* encodes a proline-rich protein that includes a putative heavy metal-binding domain and protein-protein interaction motifs. The resistant allele *pi21* carrying deletions in the proline-rich motif can reduce blast infection rate ([Fukuoka et al. 2009]). *Pik*, *Pik-m* and *Pik-p* are located at the locus of *Pik* on chromosome 11, and interestingly, each of them requires two independent NBS-LRR genes for the blast resistance ([Zhai et al. 2011];[Ashikawa et al. 2008];[Yuan et al. 2011]). Similarly, both *Pi5* and *Pia* also require two NBS-LRR members for their resistance function ([Lee et al. 2009];[Okuyama et al. 2011]).

At least eight blast resistance genes were identified from the *Pi2/9* locus, which is located on the short arm and near the centromere of chromosome 6. Among them, *Pi9*, *Pi2* and *Piz-t* were successfully cloned ([Qu et al. 2006];[Zhou et al. 2006]). *Pi26(t)* ([Wu et al. 2005]), *Pigm(t)* ([Deng et al. 2006]), *Piz(t)* ([Fjellstrom et al. 2006]), *Pi40(t)* ([Jeung et al. 2007]) and *Pi50(t)* ([Zhu et al. 2012]) are in the process of being cloned by different laboratories. Interestingly, most of them confer broad-spectrum resistance to diverse *M. oryzae* races or isolates. The near isogenic line C101A51 carrying *Pi2* is resistant to 455 isolates collected from Philippines and most of the 792 isolates from China ([Chen et al. 1996, 1999]). The *Pi9*-bearing line, 75-1-127, is resistant to 43 isolates collected from 13 different countries ([Liu et al. 2002]). *Piz-t* and *Pigm* from Toride and Gumei4, respectively, are resistant to more than 90% of tested isolates from China and Thailand ([Shen et al. 2003]). The near-isogenic line containing *Pi50(t)* is incompatible to 97.7% of the 523 isolates from different regions of China ([Zhu et al. 2012]). However, the underlying mechanism of broad-spectrum resistance of these genes is still not well understood.

Jefferson, a long-grain tropical japonica cultivar grown in the southern U.S., has retained its resistance to blast since its first use in 1997 ([McClung et al. 1997];[Skamnioti and Gurr 2009]). It was reported that Jefferson possesses three blast resistance genes, *Piz(t)*, *Pi-d(t)* and *Pi-k*^*h*^*(t)*, based on its disease reactions ([McClung et al. 1997]). Our preliminary observation showed that Jefferson was immune in the blast nursery of Taojiang County, Hunan Province, China, which contained 11 major *M. oryzae* races including ZC9, ZC11, ZE3, ZB29, ZG1, ZB25, ZB31, ZB13, ZC7, ZA9, and ZF1 (unpublished). To determine the genetic basis of broad-spectrum resistance in Jefferson, we performed greenhouse inoculations with individual isolates and genetic analysis using an F_2_ population derived from a cross between Jefferson and the susceptible cultivar CO39. We identified a dominant *R* gene in Jefferson on chromosome 6 at the *Pi2/9* locus, named *Pi2-2*. Allelism analysis indicated that *Pi2-2* is tightly linked or allelic to *Pi9.* We constructed a BAC contig in the genomic region and fine-mapped the gene within a region approximately 270 kb. These data will facilitate both the positional cloning of the *R* gene and molecular breeding programs of rice blast resistance.

## Results

### Resistance spectrum of Jefferson to 28 *M. Oryzae* isolates

To test the resistance spectrum of Jefferson, we inoculated the cultivar with 28 *M. oryzae* isolates collected from six countries, and the inoculation results are summarized in Additional file1: Table S1. Three known broad-spectrum resistant cultivars, Tianye carrying *Pi2-1* and *Pi51* ([Wang et al. 2012]), XZ3150 carrying *Pi47* and *Pi48* ([Huang et al. 2011]), and 75-1-127 carrying *Pi9* ([Qu et al. 2006]) were used as resistance controls and the highly susceptible cultivar CO39 was used as a susceptible control. Interestingly, Tianye was resistant to all the isolates and Jefferson was only susceptible to the blast isolate RB11 from Japan. XZ3150 was susceptible to three isolates (236–1, RB6 and ROR1) and 75-1-127 was susceptible to two isolates (ROR1 and X2007A-7). By contrast, the susceptible control cultivar CO39 was susceptible to 27 of all 28 tested isolates. These results indicate that Jefferson confers broad-spectrum resistance to *M. oryzae*.

### Resistance to *M. oryzae* isolate 318–2 is controlled by a single dominant locus in Jefferson

The *M. oryzae* isolate 318–2 from Hunan Province of China was used for genetic analysis of the blast resistance in Jefferson. We developed the F_2_ population derived from a cross between Jefferson and CO39. All the F_1_ plants were resistant to 318–2 (32R:0S), indicating that the dominant inheritance of the *R* gene in Jefferson. The segregation of resistant and susceptible individuals in the F_2_ population fitted a ratio of 3:1 (194R:60S, χ2=0.257, 0.5<P<0.9 ), suggesting that the resistance to 318–2 is controlled by a single dominant *R* gene in Jefferson. We designated this *R* gene in Jefferson as *Pi2-2*.

### *Pi2-2* is tightly linked or allelic to *Pi9* on chromosome 6

Previous research reported that there are three blast resistance genes, *Piz(t)*, *Pi-d(t)* and *Pi-k*^*h*^*(t)*, in Jefferson ([McClung et al. 1997]). *Piz(t)* is located on chromosome 6 near the *Pi2/9* locus ([Fjellstrom et al. 2006]). *Pi-d(t)* and *Pi-k*^*h*^*(t)* are located on chromosome 11. Therefore, we selected 25 SSR markers around the *Pi2/9* and *Pi-k*^*h*^ loci for linkage analysis. Twenty highly resistant and twenty highly susceptible individuals from the F_2_ population of the Jefferson×CO39 cross were genotyped with the polymorphic markers. No marker around the *Pi-k*^*h*^ locus co-segregated with the resistance to 318–2. But two polymorphic SSR markers around *Pi2/9*, RM7178 and RM7311 (Table1), were associated with the resistance, indicating that *Pi2-2* is located on chromosome 6.

**Table 1:** **Polymorphic SSR markers around the** ***Pi2/9*** **locus used for linkage analysis**

Markers	Forward primer (5′-3′)	Reverse primer (5′-3′)	Genomic position (bp)	Expected size (bp)
MRG5836^a^	AAAAACCTAGAAAATGGGAAAATG	TATAAGCCGCAGCCAAATTC	9308979-9309076	98
RM19817^b^	CCAAGGAGGTGATCCAGGAGTGC	CGGCAGAGCAGACGACATGG	10137012-10137394	383
RM7178^b^	CCGTGAGATGGGCTACCTAC	TAACCTTCACAGCGAACGTG	10198893-10199043	151
AP5659-5^a^	CTCCTTCAGCTGCTCCTC	TGATGACTTCCAAACGGTAG	10357166-10357453	288
AP5659-3^a^	TCTTTCCTAGGGAACCAAAG	AAGTAGTTGCTGAGCCATTG	10406597-10406825	229
RM7311^b^	CGTGGCGCCTTTAATCTC	AGTGGTCGTTGAACTCGGAG	11045702-11045848	147

^a^ Previously reported markers in this region.

^b^ SSR markers released by Gramene database (http://www.gramene.org/db/markers/).

Genomic position and expected PCR product size for each marker were determined based on the reference sequence of rice cultivar Nipponbare released by International Rice Genome Sequencing Program (IRGSP).

Previous studies showed that *Pi2* and *Piz-t* are tightly linked to *Pi9* ([Zhou et al. 2006, 2007]) and *Piz(t)* is allelic or tightly linked to *Piz-t* ([Hayashi et al. 2004]). However, the exact location of *Piz(t)* has not been determined yet. To understand the linkage relationship between *Pi2-2* and the *R* genes in the same region, we developed an F_2_ population from a cross between Jefferson and *Pi9-* carrying line 75-1-127 for allelism test. A total of 637 F_2_ individuals were inoculated with *M. oryzae* isolate 318–2, which was incompatible to both Jefferson and 75-1-127, to observe the phenotype segregation. No susceptible plant was found in 637 F_2_ individuals, suggesting that *Pi2-2* is tightly linked or allelic to the *Pi9* gene.

### Jefferson shows different resistance spectrum with the cultivars carrying other *R* genes at the *Pi2/9* locus

Previous research showed that the three cloned *R* genes at *Pi2/9* locus have different resistance spectra. 75-1-127 (*Pi9*) was susceptible to ROR1, a *M. oryzae* strain from Korea. The isolate CHNOS60-2-3 from China could distinguish C101A51 (*Pi2*) and Toride *(Piz-t)* resistance specificities ([Zhou et al. 2006]). However, Jefferson was immune to both of them (Table2). In the inoculations with 28 blast isolates (Additional file1: Table S1), Jefferson and 75-1-127 also have different resistance spectra. In addition, another two isolates from Hunan Province, China, showed different reactions to Jefferson and Toride (*Piz-t*) or 5173 (*Pi2*). These results suggest that *Pi2-2* is a different *R* gene at the *Pi2/9* locus. However, isogenic lines with all the *R* genes at the *Pi2/9* locus should be used in inoculations with different isolates to confirm the conclusion.

**Table 2:** **The disease reactions of Jefferson and donors of** ***Pi2, Piz-t*** **and** ***Pi9***

Isolates	Origin	Cultivars				
		Jefferson	5173 (***Pi2***)	Toride (***Piz-t***)	75-1-127***(Pi9)***	CO39
ROR1	Korea	R	R	R	S	S
CHNOS60-2-3	China	R	S	R	R	S
236-1	China	R	R	S	R	S
X2007A-7	China	R	R	S	S	S

R and S denote resistant and susceptible reaction, respectively.

### Fine mapping and *in silico* mapping of the *Pi2-2* gene

**Figure 1:**
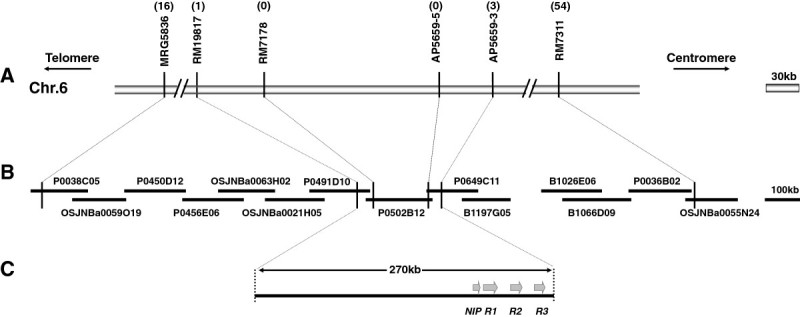
**Genetic and physical maps around the** ***Pi2-2*** **locus.** (**A**) A high resolution map of *Pi2-2*. The numbers in parentheses above the map indicate the numbers of recombinants detected in the mapping population. (**B**) A virtual contig map spanning the *Pi2-2* locus based on information of Nipponbare BACs released by RGRP (Rice Genome Research Program). (**C**) The position of the three NBS-LRR genes in the 270-kb contig. *NIP*, nitrite-induced protein. *R1*-*R3*, three putative NBS-LRR genes at *Pi2/9* locus.

To finely map the *Pi2-2* gene, another 14 SSR markers were used, and four of them exhibited polymorphism between the two parental lines (Table1). A total of 583 susceptible individuals from the Jefferson×CO39 F_2_ population were genotyped with these polymorphic markers. Finally, the *Pi2-2* gene was delimited by the closest flanking markers RM19817 and AP5659-3, with one and three recombinant events detected, respectively (Figure1A). The markers RM7178 and AP5659-5 co-segregated with *Pi2-2* in all 583 susceptible plants. The physical distance between the closest flanking markers, RM19817 and AP5659-3, was estimated to be about 270 kb according to the Nipponbare genome information in this region. A virtual contig map consisting of three overlapping Nipponbare BAC clones (P0491D10, P0502B12 and P0649C11) was constructed (Figure1B). Annotation of the corresponding genomic sequence indicates that there are three NBS-LRR genes in this region, which are paralogs of the *Pi9* gene (Figure1C).

### Construction of a BAC contig covering the *Pi2-2* locus

**Figure 2:**
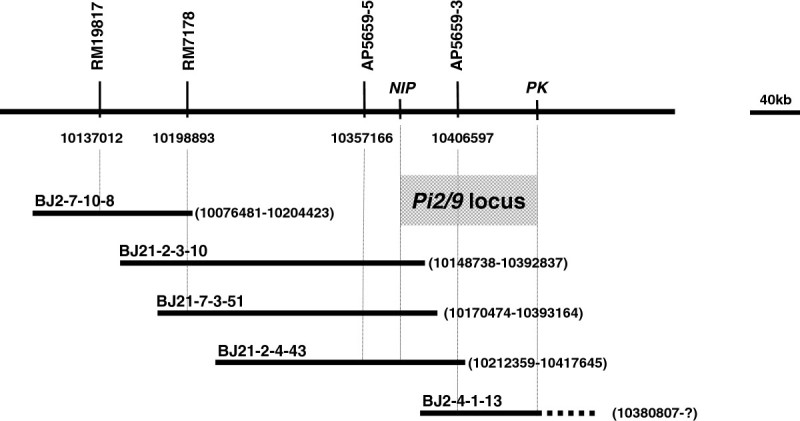
**A BAC contig map spanning the** ***Pi2-2*** **locus based on the end sequence.** The physical position of each BAC clone is shown in parentheses. *NIP*, nitrite-induced protein gene; *PK*, protein kinase gene.

For the cloning of the *Pi2-2* gene, we constructed a genomic BAC library of Jefferson with an average insert size of 140 kb. The tightly linked SSR markers spanning *Pi2-2* were used for PCR screening of the BAC library pools. Six positive clones were identified by four SSR markers and were end-sequenced (Table3). To confirm whether these BAC clones overlapped, the end sequences were compared with the corresponding sequences on chromosome 6 in the Nipponbare genome. The results showed that BAC clones BJ21-2-4-43 and BJ21-5-4-41 were the same. For clone BJ2-4-1-13, only one end was anchored at *Pi2/9* locus and no homologous sequence was identified on this chromosome compared with the Nipponbare genomic sequence for the other end. The *NIP* (nitrite-induced protein) and *PK* (protein kinase) genes are the 5′ and 3′ boundaries of the *Pi2/9* locus, respectively, these are highly conserved in different haplotypes ([Zhou et al. 2007]). Thus, the specific primer pairs NIP-2F/R (NIP-2F, 5′- TTTGGCGTGTCACATCGG-3′; NIP-2R, 5′-TGGAGCGGAGACAGAGTGG-3′) and PK-1F/R (PK-1F, 5′-CGTTCACTGACTTCCCTTTCCC-3′; PK-1R, 5′-TCCGCATCGCCGTCTTCTG-3′), designed based on the *NIP* and *PK* sequences, were employed for detecting the relative location of the five BAC clones at the *Pi2/9* locus. The PCR results showed BJ2-4-1-13 contained the *PK* gene. A contig map consisting of 5 BAC clones (BJ2-7-10-8, BJ21-2-3-10, BJ21-7-3-51, BJ21-2-4-43 and BJ2-4-1-13) was constructed that covered both *Pi2-2* and the whole *Pi2/9* locus in Jefferson (Figure2).

**Table 3:** **PCR screening of positive BAC clones from the Jefferson BAC library**

BAC clones	Markers						Physical locations	
	RM19817	RM7178	AP5659-5	NIP	AP5659-3	PK	Start	Stop
BJ2-7-10-8	+	+	-	-	-	-	10076481	10204423
BJ21-2-3-10	-	+	+	+	-	-	10148738	10392837
BJ21-7-3-51	-	+	+	+	-	-	10170474	10393164
BJ21-2-4-43	-	-	+	+	+	-	10212052	10417645
BJ21-5-4-41	-	-	+	+	+	-	10212052	10417645
BJ2-4-1-13	-	-	-	-	+	+	10380807	N/A

+, positive; –, negative.

The physical locations were determined by comparing the end sequences of the BAC clones with the Nipponbare genomic sequence.

## Discussion

Many plant disease resistance genes are located in complex clusters in which multiple copies of closely related sequences are formed through gene duplication and uneven crossing over. Allelic genes in different genetic backgrounds have evolved to carry diverse resistance specificities due to exposure of these loci to different pathogen populations. In rice, over half of the identified blast resistance genes are clustered at different loci, especially on chromosomes 6, 11 and 12. The *Pi2/9* locus is a region with at least eight *R* genes ([Yang et al. 2009];[Zhu et al. 2012]), and contains several NBS-LRR type genes in both cultivated and wild rice lines ([Zhou et al. 2007];[Dai et al. 2010]). Three *R* genes at this locus have been successfully isolated. The paralog *NBS2-Pi9* is the *Pi9* gene, and the paralogs *NBS4-Pi2* and *NBS4-Piz-t* are the *Pi2* and *Piz-t* genes, respectively ([Zhou et al. 2006]). In our study, three candidate NBS-LRR genes (NBS-LRR1, NBS-LRR2 and NBS-LRR3) at the *Pi2/9* locus were identified for *Pi2-2* according to the sequence of Nipponbare genome. However, the Nipponbare genome did not fully reflect the structure of the *Pi2-2* locus in Jefferson. Thus, sequence analysis of the BAC clones of Jefferson covering *Pi2-2* and complementation test of candidate genes are necessary for determining which NBS-LRR gene is *Pi2-2.*

Three blast resistance genes, *Piz(t)*, *Pi-d(t)* and *Pik-*^*h*^*(t)*, were reported in Jefferson ([McClung et al. 1997]). *Pi-d(t)* and *Pik-*^*h*^*(t)* are tightly linked on chromosome 11. *Piz(t)* was originally reported in the U.S. rice cultivar Zenith ([Kiyosawa 1967]), and has been widely introduced into different cultivars by rice breeders ([Conaway-Bormans et al. 2003]). *Piz(t)* was mapped on the short arm of chromosome 6, close to the centromere, by several groups using different cultivars ([Hayashi et al. 2006];[Fjellstrom et al. 2006];[Conaway-Bormans et al. 2003]), but the exact location has not been determined yet. Based on the fine mapping results in this study, we speculate that *Pi2-2* is likely *Piz(t)*. Our on-going cloning effort of the *Pi2-2* gene will provide us the answer in the near future.

## Conclusions

This study demonstrated that the rice cultivar Jefferson harbors the blast resistance gene *Pi2-2* at the *Pi2/9* locus on chromosome 6. The gene was finely mapped to a 270 kb interval. A BAC contig covering *Pi2-2* was constructed, which provides essential foundation for the isolation of the *R* gene.

## Methods

### Plant materials

Seven rice cultivars, Jefferson, Tianye, XZ3150, 5173 (*Pi2*), Toride (*Piz-t*), 75-1-127 (*Pi9*) and CO39, were used in this study. F_1_ and F_2_ populations from a cross between Jefferson and highly susceptible cultivar CO39 were constructed for genetic analysis. The F_2_ population derived from a cross between Jefferson and 75-1-127 was constructed for allelism tests.

### Blast inoculation and disease evaluation

The 28 *M. oryzae* isolates used in the study are listed in Additional file1: Table S1. The collection sites and providers are included in the table. Rice seedlings at 3–4 leaf-stage were spray-inoculated with *M. oryzae* spore suspensions (1.5×10^5^ spores/ml) and then kept in darkness at 25°C-27°C and over 90% relative humidity for 24 h. The inoculated plants were subsequently kept under a 12/12 (day/night) photoperiod at the same temperature and relative humidity. Disease reaction evaluation was carried out 7 days after inoculation according to the 0–5 scoring system described by[(Bonman et al. 1986)].

### Genetic and allelism analysis

The Jefferson×CO39 F_2_ population was inoculated with the *M. oryzae* isolate 318–2, which is avirulent to Jefferson and virulent to CO39. 318–2, which is also avirulent to 75-1-127, was employed to inoculate the Jefferson×75-1-127 F_2_ population for allelism analysis.

### Genotyping and genetic mapping

A total of 39 SSR markers spanning the *Pi2/9* and *Pik* loci were used for the polymorphism survey between Jefferson and CO39. Six polymorphic SSR markers spanning the *Pi2/9* locus were used for preliminary and fine mapping of the *R* gene in Jefferson (Table1). The genomic DNA of 20 highly resistant and 20 susceptible F_2_ individuals, which were phenotypically confirmed in the F_3_ generation, were extracted from leaves for segregation analysis ([Saghai-Maroof et al. 1984]). All PCRs began with a denaturation step of 94°C/4 min, followed by 35 cycles of (A) 94°C/30 sec, 55°C/30 sec, 72°C/30 sec, with a final extension step of 72°C/7 min. Linkage analysis was performed using the MAPMAKER/V3.0 using all highly susceptible individuals.

### Physical mapping of the *Pi2-2* locus

The physical positions of the markers tightly linked to *Pi2-2* locus were determined based on the genome of Nipponbare using the BLAST program on Gramene (http://www.gramene.org/Multi/blastview) ([Jaiswal et al. 2006]). The genomic sequences flanked by the markers RM19817 and AP5659-3 were annotated using the Rice Genome Annotation Project (http://rice.plantbiology.msu.edu/) ([Ouyang et al. 2007]) and Rice Genome Automated Annotation System (http://ricegaas.dna.affrc.go.jp/) ([Sakata et al. 2002]).

### Construction of the BAC library of Jefferson

The genomic BAC library of Jefferson was constructed using the method described by[(Luo and Wing 2003)]. The markers tightly linked to *Pi2-2* were used for screening of positive clones from the BAC pools. The contig map spanning the *Pi2-2* locus was constructed based on the end sequencing results of the positive BAC clones.

## Electronic supplementary material

Additional file 1:**Table S1.** Disease reaction of Jefferson and other 4 cultivars to 28 M. oryzaeisolates collected from different regions. (DOC 203 KB)

Below are the links to the authors’ original submitted files for images.

Authors’ original file for figure 1

Authors’ original file for figure 2
