# Supplementary material for: Molecular mapping of the Pi2/9 allelic gene Pi2-2 conferring broad-spectrum resistance to Magnaporthe oryzae in the rice cultivar Jefferson
Source: Rice (N Y). 2012 Oct 3;5:29. doi: 10.1186/1939-8433-5-29 (PMC5520841; doi:10.1186/1939-8433-5-29)
Supplement: Supplementary file 1 — Additional file 1:Table S1. Disease reaction of Jefferson and other 4 cultivars to 28 M. oryzaeisolates collected from different regions. (DOC 203 KB) [file 12284_2012_21_MOESM1_ESM.doc]

Supplemental Table 1. Disease reaction of Jefferson and other 4 cultivars to 28 *M. oryzae* isolates collected from different regions

| Isolates | Source | Cultivars | | | | | Providers |
| --- | --- | --- | --- | --- | --- | --- | --- |
|  |  | Jefferson | Tianye | XZ3150 | 75-1-127 | CO39 |  |
| 318-2 | HN | R | R | R | R | S | Erming Liu^a^ |
| 110-2 | HN | R | R | R | R | S | Erming Liu |
| 8 7-4 | HN | R | R | R | R | S | Erming Liu |
| 193-1-1 | HN | R | R | R | R | S | Erming Liu |
| 220-1-1 | HN | R | R | R | R | S | Erming Liu |
| 107-2 | HN | R | R | R | R | S | Erming Liu |
| 236-1 | HN | R | R | S | R | S | Erming Liu |
| CHL438 | HN | R | R | R | R | S | Qinghua Pan^b^ |
| CHL440 | HN | R | R | R | R | S | Qinghua Pan |
| CHL471 | HN | R | R | R | R | S | Qinghua Pan |
| X2007A-7 | HN | R | R | R | S | S | Zonghua Wang^c^ |
| RB1 | GD | R | R | R | R | R | Youliang Peng^d^ |
| RB2 | GD | R | R | R | R | S | Youliang Peng |
| RB6 | GD | R | R | S | R | S | Youliang Peng |
| RB7 | GD | R | R | R | R | S | Youliang Peng |
| RB9 | GD | R | R | R | R | S | Youliang Peng |
| CHL1743 | GD | R | R | R | R | S | Qinghua Pan |
| RB16 | FJ | R | R | R | R | S | Youliang Peng |
| RB18 | FJ | R | R | R | R | S | Youliang Peng |
| RB19 | FJ | R | R | R | R | S | Youliang Peng |
| RB20 | FJ | R | R | R | R | S | Youliang Peng |
| CHL506 | FJ | R | R | R | R | S | Qinghua Pan |
| KJ201 | South Korea | R | R | R | R | S | Yong-Hwa Lee^e^ |
| ROR1 | South Korea | R | R | S | S | S | J.H. Roh^f^ |
| PO6-6 | Philippines | R | R | R | R | S | Hei Leung^g^ |
| RB11 | Japan | S | R | R | R | S | Youliang Peng |
| IC-17 | USA | R | R | R | R | S | Yulin Jia^h^ |
| RB21 | France | R | R | R | R | S | Youliang Peng |

HN, GD and FJ stand for Hunan, Guangdong and Fujian Province of China, respectively

R and S denote for resistant and susceptible reaction, respectively

^a^ Dr. Erming Liu (Hunan Agricultural University, China)

^b^ Dr. Qinghua Pan (South China Agricultural University, China)

^c^ Dr. Zonghua Wang (Fujian Agriculture and Forestry University, China)

^d^ Dr. Youliang Peng (China Agricultural University, China)

^e^ Dr. Yong-Hwa Lee (Seoul National University, Republic of Korea)

^f^ Dr. J.H. Roh (National Institute of Crop Science, RDA, Republic of Korea)

^g^ Dr. Hei Leung (International Rice Research Institute, Philippines)

^h^ Dr. Yulin Jia (USDA National Rice Research Center, Arkansas, USA**)**
