# Supplementary figures and images for: Molecular mapping of the Pi2/9 allelic gene Pi2-2 conferring broad-spectrum resistance to Magnaporthe oryzae in the rice cultivar Jefferson
Source: Rice (N Y). 2012 Oct 3;5:29. doi: 10.1186/1939-8433-5-29 (PMC5520841; doi:10.1186/1939-8433-5-29)

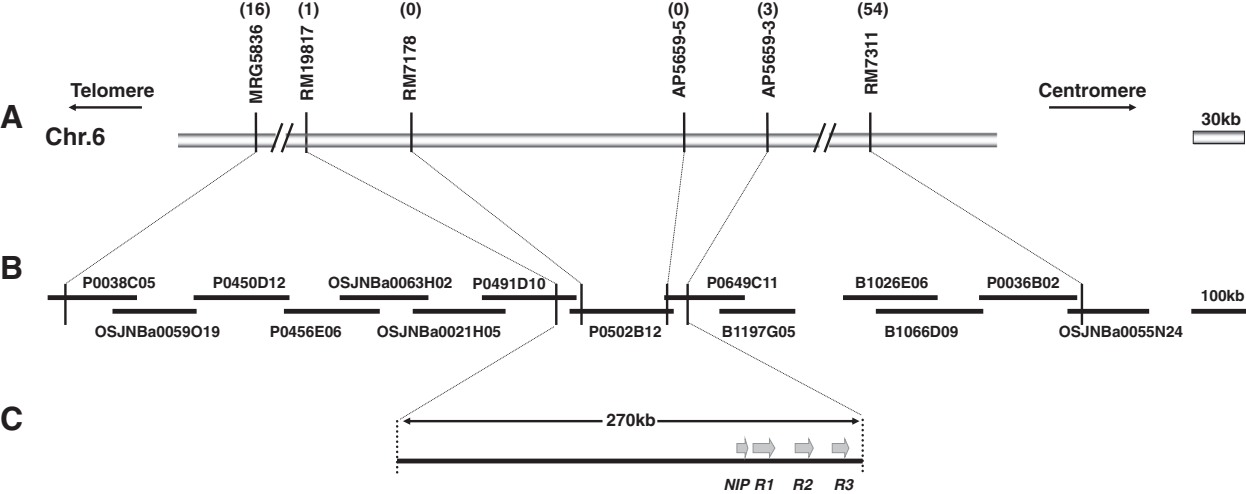

Supplement: Supplementary file 2 — Authors’ original file for figure 1 [file 12284_2012_21_MOESM2_ESM.pdf]

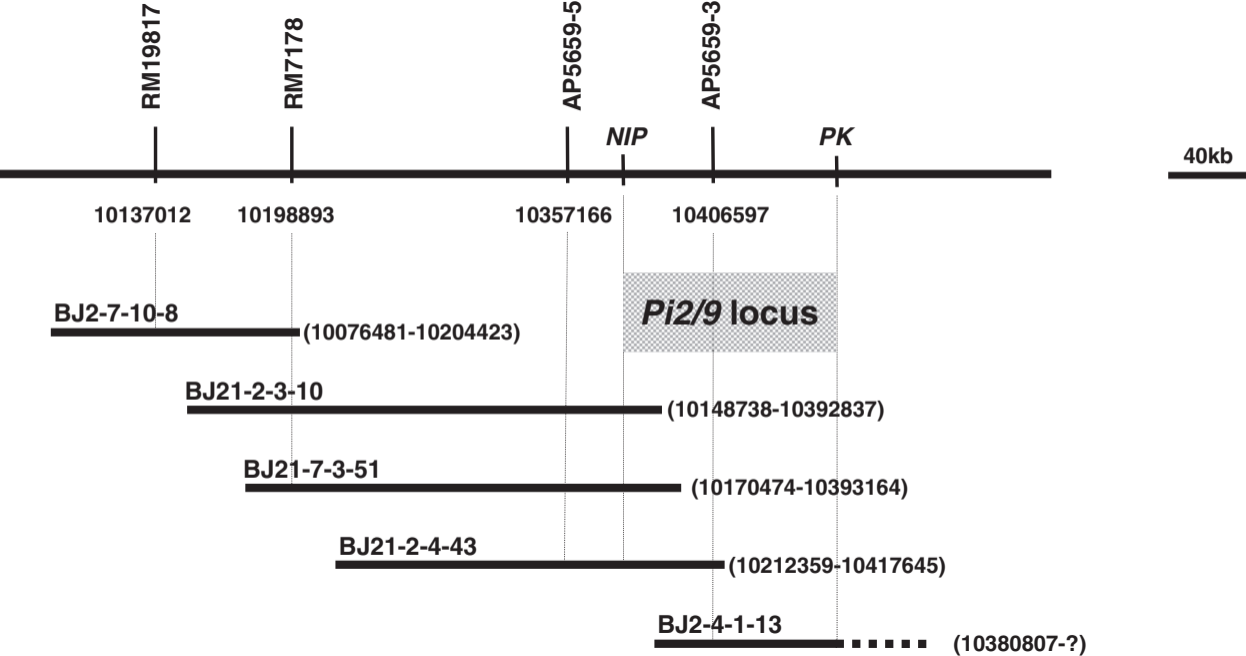

Supplement: Supplementary file 3 — Authors’ original file for figure 2 [file 12284_2012_21_MOESM3_ESM.pdf]
